# Supplementary material for: Evaluation of Plant Ceramide Species-Induced Exosome Release from Neuronal Cells and Exosome Loading Using Deuterium Chemistry
Source: Int J Mol Sci. 2022 Sep 15;23(18):10751. doi: 10.3390/ijms231810751 (PMC9505575; doi:10.3390/ijms231810751)
Supplement: Supplementary file 1 [file ijms-23-10751-s001.zip › ijms-1916273-supplementary.pdf]

Supplementary Material

Evaluation of Plant Ceramide Species-induced Exosome Release from Neuronal Cells and Exosome Loading Using Deuterium Chemistry

Yuta Yuta Murai [1,2,*], Takumi Honda [1], Kohei Yuyama [3,*], Daisuke Mikami [3], Koichi Eguchi [4], Yuichi Ukawa [5], Seigo Usuki [3], Yasuyuki Igarashi [3] and Kenji Monde [1,2,*]

1 Graduate School of Life Science, Hokkaido University, Kita 21 Nishi 11, Sapporo 001-0021, Japan

2 Faculty of Advanced Life Science, Hokkaido University, Kita 21 Nishi 11, Sapporo 001-0021, Japan

3 Lipid Biofunction Section, Faculty of Advanced Life Science, Hokkaido University, Kita-21, Nishi-11, Kita-ku, Sapporo 001-0021, Japan

4 Innovation and Business Development Headquarters, Daicel Corporation, Myoko, Niigata 944-8550, Japan

5 Healthcare SBU Business Strategy, Daicel Corporation, Tokyo 108-8259, Japan; yi_ukawa@jp.daicel.com

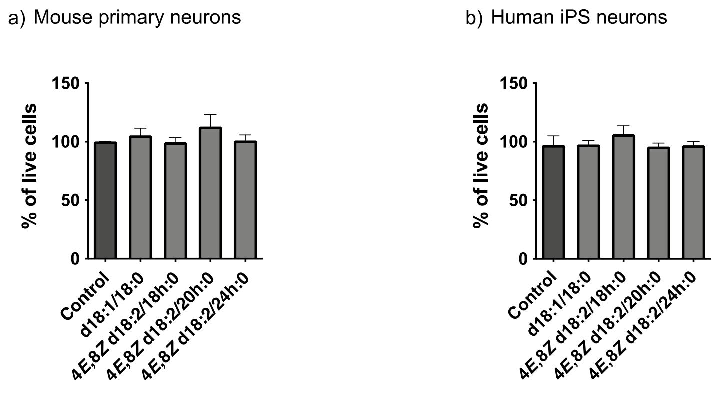

Supplementally Figure S1. Cell Counting Kit-8 (CCK-8) cell viability assays of primary mouse neurons and human iPS neurons exposed to the indicated Cers at 10 μM for 24 h. Results are normalized against controls and are represented as the mean ± S.D.
